# Forward Behavioral Modeling of a Three-Way Amplitude Modulator-Based Transmitter Using an Augmented Memory Polynomial

**DOI:** 10.3390/s18030770

**Published:** 2018-03-03

**Authors:** Jatin Chatrath, Mohsin Aziz, Mohamed Helaoui

**Affiliations:** iRadio Lab, Department of Electrical and Computer Engineering, University of Calgary, 2500 University Dr. NW, Calgary, AB T2N 1N4, Canada; azizm@ucalgary.ca (M.A.); mhelaoui@ucalgary.ca (M.H.)

**Keywords:** AM-AM, AM-PM, amplitude modulator, behavioral modeling, mixer-less transmitters, NMSE, variable gain amplifiers

## Abstract

Reconfigurable and multi-standard RF front-ends for wireless communication and sensor networks have gained importance as building blocks for the Internet of Things. Simpler and highly-efficient transmitter architectures, which can transmit better quality signals with reduced impairments, are an important step in this direction. In this regard, mixer-less transmitter architecture, namely, the three-way amplitude modulator-based transmitter, avoids the use of imperfect mixers and frequency up-converters, and their resulting distortions, leading to an improved signal quality. In this work, an augmented memory polynomial-based model for the behavioral modeling of such mixer-less transmitter architecture is proposed. Extensive simulations and measurements have been carried out in order to validate the accuracy of the proposed modeling strategy. The performance of the proposed model is evaluated using normalized mean square error (NMSE) for long-term evolution (LTE) signals. NMSE for a LTE signal of 1.4 MHz bandwidth with 100,000 samples for digital combining and analog combining are recorded as −36.41 dB and −36.9 dB, respectively. Similarly, for a 5 MHz signal the proposed models achieves −31.93 dB and −32.08 dB NMSE using digital and analog combining, respectively. For further validation of the proposed model, amplitude-to-amplitude (AM-AM), amplitude-to-phase (AM-PM), and the spectral response of the modeled and measured data are plotted, reasonably meeting the desired modeling criteria.

## 1. Introduction

The transmitter is the key block in any wireless communication and sensor network. It performs the functions of digital modulation, frequency up-conversion, and amplification of the signal before transmitting it through the antenna to a remote receiver. Various transmitter topologies have been proposed in the literature, such as super-heterodyne, direct conversion, and low IF [1,2,3,4], with the aim of improving the quality of the transmitted signal. The direct conversion transmitter is the most commonly used topology due to the ease of implementation and its simplicity in architecture as it only uses one frequency up-conversion stage as compared to the aforementioned counterparts. 

In order to cater to the needs of different standards, the evolution of modern communication signals has ignited the quest for multi-standard transmitters. Thus, reconfigurabilty, integration, and cost efficiency are the prime parameters to develop an ideal software-defined radio (SDR) [5]. Multi-standard signals are subject to various distortions when passed through different stages of the transmitter due to the imperfections in the various components present in the transmitter. Several block-based behavioral models, such as Hammerstein–Wiener, augmented Weiner, augmented Hammerstein, and memory polynomial, are proposed for modeling the non-linear distortions in the transmitters [6,7,8,9,10,11,12] introduced by the power amplifiers during the amplification of the signal. 

These methods, however, do not consider the impairments introduced by the modulator, such as in-phase and quadrature-phase (I/Q) imbalance and DC offset, and only mitigate the non-linear distortions introduced by power amplifiers (PAs). Apparently, in the literature, several models such as, Volterra series-based model, neural network-based models, and variations of memory polynomial models, have been proposed, which successfully model and mitigate the impairments introduced by modulator and power amplifiers [13,14,15,16,17,18]. However, this comes at the cost of complexity and higher processing rates.

**Figure 1:**
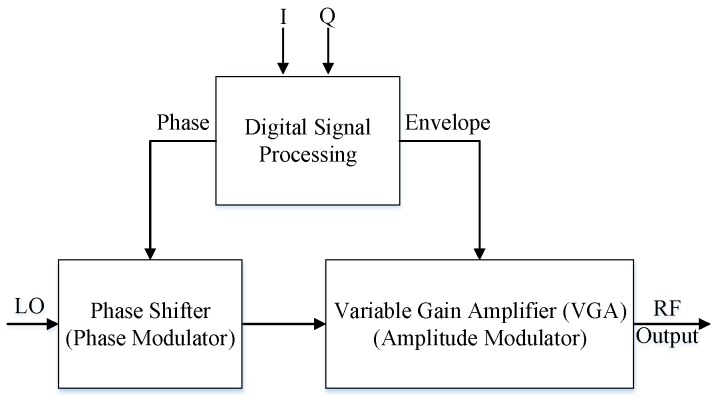
Block diagram of the mixer-less polar transmitter.

Evidently, impairments introduced by mixers are critical and need to be catered. Transmitter architecture would be much simpler if the use of mixers is avoided altogether. Recently, in order to avoid the impairments of the modulator, a new mixer-less polar transmitter topology was proposed [19]. The mixer-less polar transmitter, as shown in Figure 1, is implemented using a variable gain amplifier (VGA) and a phase shifter. The envelope signal is generated digitally at the baseband and fed to the gain control input pin of the VGA, which recreates the envelope signal at the RF output, while the phase shifter translates the baseband signal to RF. Ultimately, the phase-modulated RF signal is combined with the envelope of the VGA. Mixer-less polar transmitters have various advantages over conventional transmitters, such as the omission of bulky filters and reduced complexity. However, the phase shifter has issues with noise and affects the quality of the RF signal produced at its output. Additionally, as suggested in [20], the phase variations exhibited by the phase shifter when driven with a constant voltage could not be modeled and compensated for.

Later, the mixer-less branch-by-branch three-way amplitude modulator-based transmitter was proposed [20]. This topology avoided the use of a phase shifter and, thus, used three VGAs as envelope modulators. A complex envelope of the signal was decomposed into three envelope components using a three-way decomposition algorithm. The VGAs translated these three baseband components to the carrier frequency. The three envelopes produced at the output of the VGAs were then combined digitally before being transmitted. The method proposed in [20] also provides a reverse behavioral model for the proposed architecture. The authors employ a memory polynomial model for each branch of the transmitter to linearize each of its three branches. However, there are certain drawbacks associated with such a modeling strategy, such as both the individual input and output of each branch are required to model the system. This, in practice, is not effective since the splitter and combiner are embedded and have to be removed to gain access to individual inputs/outputs. Additionally, time delay and phase adjustments have to be performed individually in each branch, making the process laborious and complex as separate time adjustment routines have to be performed for each branch of the transmitter. Moreover, [20] reports the use of a digital splitter and combiner, which is not close to a practical system implementation. 

These disadvantages, however, can be mitigated if a model of the complete mixer-less three-way amplitude modulator-based transmitter using analog splitters and combiners is used, thus imitating an actual system. This forms the motivation behind the proposed solution [21]. In order to model the magnitude and phase non-linearities introduced in all the three braches by three VGAs in a single block, a new augmented memory polynomial model is proposed. The performance of the modified memory polynomial is tested using different bandwidths of the long-term evolution (LTE) signal. 

This paper is organized as follows: Section 2 introduces the mixer-less three-way amplitude modulator-based transmitter and proposed decomposition algorithm; Section 3 details the proposed forward behavioral model for the mixer-less three-way transmitter architecture; Section 4 describes the implementation of the transmitter architecture with analog and digital combining; Section 5 presents the measurement results; and Section 6 concludes the paper.

## 2. The Mixer-Less Three-Way Amplitude Modulator-Based Transmitter Architecture 

### 2.1. The Mixer-Less Three-Way Amplitude Modulator-Based Transmitter

**Figure 2:**
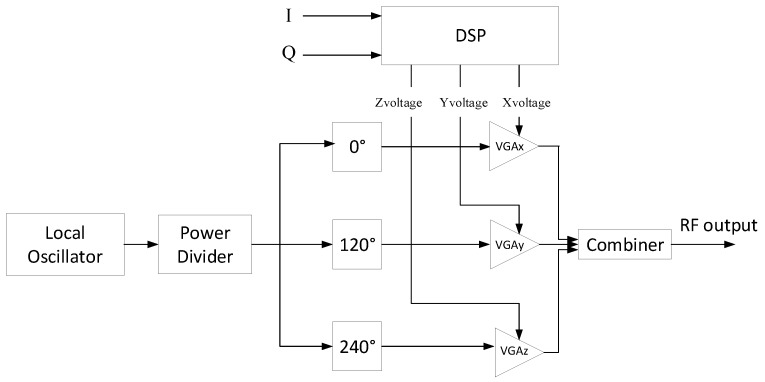
High-level block schematic of the mixer-less three-way amplitude modulator-based transmitter.

The high level schematic diagram of the three-way transmitter architecture is shown in Figure 2. It consists of three VGAs which work as envelope modulators, as explained in [20]. The local oscillator (LO) signal is fed to a three-way power divider. The three outputs of the power divider are further rotated by 0°, 120°, and 240° using phase shifters. The 0° LO phase-shifted signal is fed to the RF input port of VGAx, the 120° phase rotated LO signal is fed to the RF input port of VGAy, and the 240° phase-shifted LO signal is fed to the RF input port of VGAz. Xvoltage, Yvoltage, and Zvoltage are control voltages which are generated in the digital signal processor (DSP) by decomposing the LTE signal and mapping them into voltages. These control voltages act as amplitude modulating signals to the LO. Amplitude-modulated signals are obtained at the RF output port of each VGA, which is further combined by a three-way power combiner to produce the complex RF output signal. The passive components, such as the power combiner/divider and phase shifters, used are broadband. Moreover, VGAs operate over a wide RF bandwidth and do not have any spurious emissions; hence, no filtering is required in this distinct transmitter architecture. In a nutshell, this transmitter translates the baseband I/Q signal to RF without using mixers, filters, phase modulators, and up-convertor circuits.

### 2.2. Signal Decomposition (Three Coordinate)

A complex I/Q signal can be represented in polar format (*r*, *θ*) as:

(1) $$r(n)=\sqrt{I{\left(n\right)}^{2}+Q{\left(n\right)}^{2}}$$

(2) $$\theta (n)=\tan^{-1}\left(\frac{Q(n)}{I(n)}\right)$$

where *r*(*n*) and *θ*(*n*) are the magnitude and phase of the signal, respectively. A signal of the form *S*_in_(*n*) = *r*(*n*)*e^jθ^*^(*n*)^ can be decomposed into three positive real components *x*(*n*), *y*(*n*), and *z*(*n*) with a phase difference of 120° between them according to the law of sines, as suggested in [20]. From here on, the sample index *n* has been removed for simplicity. For different values of *θ*, *S*_in_ can be decomposed into *x*, *y*, and *z* components. Here, *x* is a vector that consists of *x*_in1_, *x*_in2_, and *x*_in3_ based on the coordinates in which the present sample lies. For different values of *θ*, *S*_in_ can be decomposed into *x*, *y*, and *z* components such that:

(3) $$S_{\mathrm{in}}=x_{\mathrm{in},1}+y_{\mathrm{in},1}e^{j120^{\circ }}+z_{\mathrm{in},1}e^{j240^{\circ }}$$

When 0° < *θ* < 120°, *S*_in_ can be decomposed as:

(4) $$x_{\mathrm{in},1}=A_{1}S_{\mathrm{in}}+B_{1}S_{\mathrm{in}}e^{j120^{\circ }}+C_{1}S_{\mathrm{in}}e^{j240^{\circ }}$$

(5) $$y_{\mathrm{in},1}=A_{2}S_{\mathrm{in}}+B_{2}S_{\mathrm{in}}e^{j120^{\circ }}+C_{2}S_{\mathrm{in}}e^{j240^{\circ }}$$

(6) $$z_{\mathrm{in},1}=0$$

where values of *A*_1_, *B*_1_, *C*_1_, *A*_2_, *B*_2_, and *C*_2_ are depicted in Table 1. Note that the $z_{\mathrm{in},1}$ component is zero in this coordinate as the signal lying in this sector will only have the *x*- and *y*-components and, hence, the coefficients related to $z_{\mathrm{in},1}$ are zero, as shown in Table 1. 

Similar decomposition can be achieved for other ranges of *θ*. When 120° < *θ* < 240°, *S*_in_ can be decomposed as:

(7) $$x_{\mathrm{in},1}=0$$

(8) $$y_{\mathrm{in},1}=A_{5}S_{\mathrm{in}}+B_{5}S_{\mathrm{in}}e^{j120^{\circ }}+C_{5}S_{\mathrm{in}}e^{j240^{\circ }}$$

(9) $$z_{\mathrm{in},1}=A_{6}S_{\mathrm{in}}+B_{6}S_{\mathrm{in}}e^{j120^{\circ }}+C_{6}S_{\mathrm{in}}e^{j240^{\circ }}$$

where values of *A*_5_, *B*_5_, *C*_5_, *A*_6_, *B*_6_, and *C*_6_ are depicted in Table 2. In this coordinate, the $x_{\mathrm{in},1}$ component and its corresponding coefficients are zero as the signal lying in this sector will only have the *y*- and *z*-components. 

Similarly, when 240° < *θ* < 360°, *S*_in_ can be decomposed as:

(10) $$x_{\mathrm{in},1}=A_{7}S_{\mathrm{in}}+B_{7}S_{\mathrm{in}}e^{j120^{\circ }}+C_{7}S_{\mathrm{in}}e^{j240^{\circ }}$$

(11) $$y_{\mathrm{in},1}=0$$

(12) $$z_{\mathrm{in},1}=A_{9}S_{\mathrm{in}}+B_{9}S_{\mathrm{in}}e^{j120^{\circ }}+C_{9}S_{\mathrm{in}}e^{j240^{\circ }}$$

where the values of *A*_7_, *B*_7_, *C*_7_, *A*_9_, *B*_9_, and *C*_9_ are depicted in Table 3 and $y_{\mathrm{in},1}$ and its coefficients are zero as the signal lying in this sector will only have the *x*- and *z*-components. 

In order to map *x*_in,1_, *y*_in,1_ and *z*_in,1_ components into control voltages, the following expression is used:

(13) $$X_{voltage}=20\cdot a\cdot \log_{10}(x_{\mathrm{in},1})+b$$

where *a* and *b* are constants acquired from DC voltage gain response of the VGA. Similarly, control voltages *Y*_voltage_ and *Z*_voltage_ can be obtained from similar equations to the above with different values for the constants *a* and *b*. 

**Figure 3:**
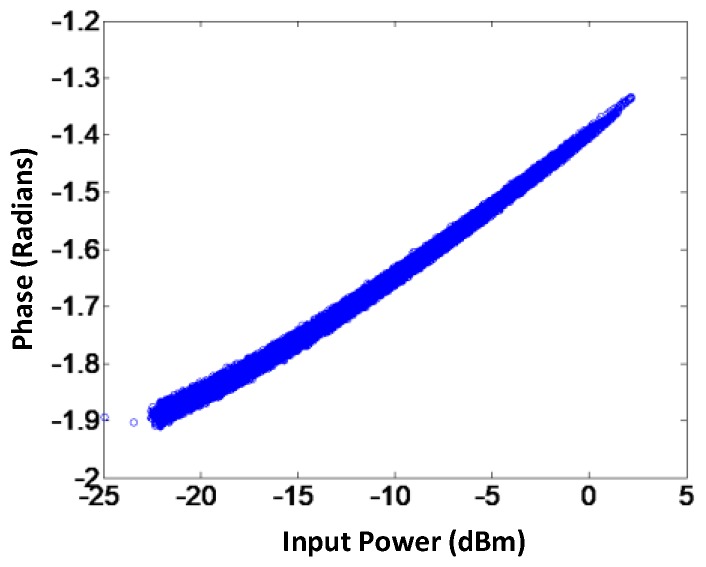
Phase response of the VGA.

**Figure 4:**
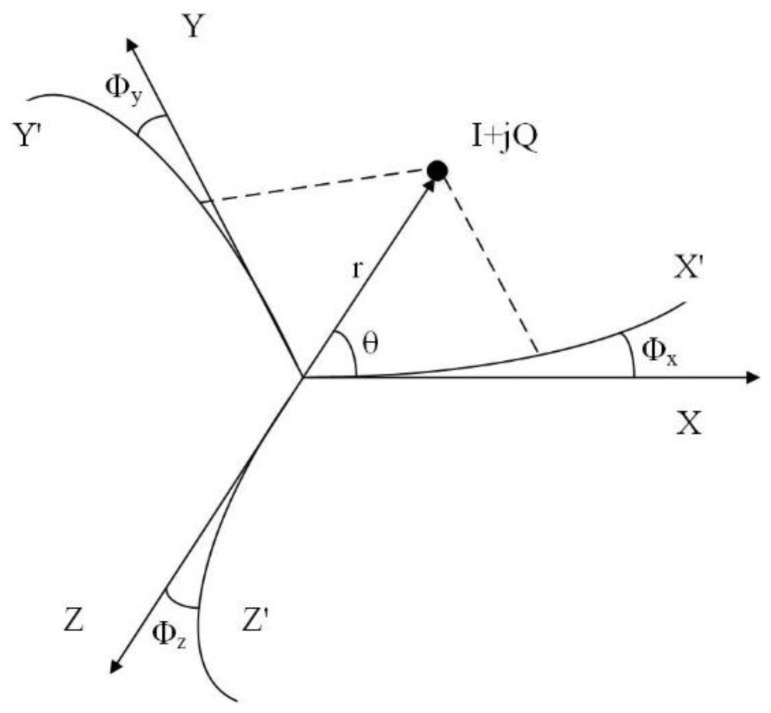
Signal decomposition based on the phase response of the VGA.

Apparently, the phase variation at the output of the VGA as a function of the gain control voltage can be seen in Figure 3. The plot shows that as the LO signal propagates through the VGA, its phase varies. Thus, at the RF output of VGAx, VGAy, and VGAz, the phases of the different components *x*, *y*, and *z* will be affected by the phase response of the VGA. Ideally, at the output of VGAx, VGAy, and VGAz the phases of the components are expected to be 0°, 120°, and 240°, respectively. Although, as seen from Figure 3, the variation in the gain control voltage affects the phase at the RF output of the VGA. For the *i*th sample, the phase of the component *x*, *y*, and *z* at the output of VGAx, VGAy, and VGAz can be represented as 0° + *Φ*_x_(*i*), 120° + *Φ*_y_(*i*), and 240° + *Φ*_z_(*i*), respectively. *Φ*_x_(*i*), *Φ*_y_(*i*), and *Φ*_z_(*i*) are the phase errors for *i*th sample in degrees introduced by VGAx, VGAy, and VGAz, respectively. To compensate for these phase errors, the complex point *I* + *jQ* is decomposed into new components along the 0° + *Φ*_x_(*i*), 120° + *Φ*_y_(*i*), and 240° + *Φ*_z_(*i*) axes as shown in Figure 4. As seen from the figure, the new axes are *X*′, *Y*′, and *Z*′. Based on the new axes and the law of sines, which is explained in [20], any complex *I* + *jQ* with magnitude *r* and angle *θ* can be decomposed into new components *X*′, *Y*′, and *Z*′.

## 3. Forward Behavioral Model for the Mixer-Less Three-Way Amplitude Modulator-Based Transmitter

As discussed in the previous section, the three-way transmitter architecture consists of three VGAs. Each VGA has a gain and phase response which needs to be modeled accurately. The method proposed in [20] provides the modeling of dynamic non-linear gain and phase responses of the single VGA using a memory polynomial model corresponding to each branch. In order to apply memory polynomials individually, it is of extreme importance to have access to all three inputs and outputs of the VGAs, namely, VGAx, VGAy, and VGAz. However, as mentioned earlier, this solution is not feasible for practical systems as the output of all the VGAs is combined digitally, whereas, in actual systems, splitting and combining are carried out via analog splitters and combiners in the RF domain. 

In this work, in order to model the mixer-less three-way amplitude modulator-based transmitter a new black box modified memory polynomial model is proposed. The output of a single VGA (e.g., VGAx) can be represented in the following manner:

(14) $$x_{\mathrm{out}}(n)=\sum_{k=1}^{K_{x}}\sum_{m=0}^{M_{x}}h_{k,m}^{x}{\left(x_{\mathrm{in},1}(n-m)\right)}^{k}$$

where $h_{k,m}^{x}$ are the complex model coefficients, *x*_out_ is the output of the VGAx, while *x*_in,1_ is the input to the model. *K_x_* and *M_x_* are the non-linearity order and memory depth, respectively. A model of VGAy and VGAz can be mathematically represented in a similar fashion. From the previous section, we can deduce that the value of *x*_in,1_, *y*_in,1_ and *z*_in,1_ can be represented as:

(15) $$x_{\mathrm{in},1}=A_{1}S_{\mathrm{in}}+B_{1}S_{\mathrm{in}}e^{j120{}^{\circ}}+C_{1}S_{\mathrm{in}}e^{j240{}^{\circ}}$$

(16) $$y_{\mathrm{in},1}=A_{2}S_{\mathrm{in}}+B_{2}S_{\mathrm{in}}e^{j120{}^{\circ}}+C_{2}S_{\mathrm{in}}e^{j240{}^{\circ}}$$

(17) $$z_{\mathrm{in},1}=A_{3}S_{\mathrm{in}}+B_{3}S_{\mathrm{in}}e^{j120{}^{\circ}}+C_{3}S_{\mathrm{in}}e^{j240{}^{\circ}}$$

where *A*_1_, *B*_1_, *C*_1_, *A*_2_, *B*_2_, *C*_2_, *A*_3_, *B*_3_, and *C*_3_ are the constants whose values variy according to the co-ordinates (specified by *θ*) and *S*_in_ is the complex input signal. The value of *x*_in,1_ is applied in Equation (14), which is followed by the application of binomial theorem expansion and other mathematical operations. Ultimately, we deduce the modified memory polynomial for 0° < *θ* < 120°, 120° < *θ* < 240°, and 240° < *θ* < 360°, respectively, as:

(18) $$S_{\mathrm{out}}(n)=\sum_{k=1}^{K}\sum_{m=0}^{M}\sum_{p_{1}+p_{2}=3k}G_{k,m,p_{1},p_{2}}\frac{\sin^{p_{1}}(\theta )\sin^{p_{2}}(\theta -120{}^{\circ})}{\sin^{3}(\theta -120{}^{\circ})-\sin^{3}(\theta )}S_{\mathrm{in}}{}^{k}(n-m);\;0\leq \theta \leq 120{}^{\circ}$$

(19) $$S_{\mathrm{out}}(n)=\sum_{k=1}^{K}\sum_{m=0}^{M}\sum_{p_{1}+p_{2}=3k}G_{k,m,p_{1},p_{2}}^{\prime }\frac{\sin^{p_{1}}(\theta -120{}^{\circ})\sin^{p_{2}}(\theta -240{}^{\circ})}{\sin^{3}(\theta -240{}^{\circ})-\sin^{3}(\theta -120{}^{\circ})}S_{\mathrm{in}}{}^{k}(n-m);\;120{}^{\circ}\leq \theta \leq 240{}^{\circ}$$

(20) $$S_{\mathrm{out}}(n)=\sum_{k=1}^{K}\sum_{m=0}^{M}\sum_{p_{1}+p_{2}=3k}G_{k,m,p_{1},p_{2}}^{\prime \prime }\frac{\sin^{p_{1}}(\theta -240{}^{\circ})\sin^{p_{2}}(\theta -360{}^{\circ})}{\sin^{3}(\theta -360{}^{\circ})-\sin^{3}(\theta -240{}^{\circ})}S_{\mathrm{in}}{}^{k}(n-m);\;240{}^{\circ}\leq \theta \leq 360{}^{\circ}$$

where $G_{k,m,p_{1},p_{2}}$, $G_{k,m,p_{1},p_{2}}^{\prime }$, and $G_{k,m,p_{1},p_{2}}^{\prime \prime }$ are the complex model coefficients, *K* and *M* are the non-linearity order and memory depth, respectively, *S*_out_(*n*) is the output of the model, and *S*_in_(*n*) is the complex input. The modeling coefficients can be obtained using least squares [12]. 

**Figure 5:**
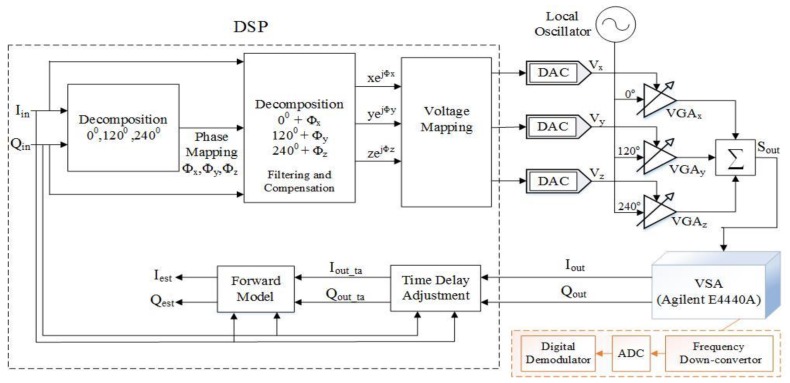
Block schematic of mixer-less three-way amplitude modulator-based transmitter, analog combining architecture with signal processing.

**Figure 6:**
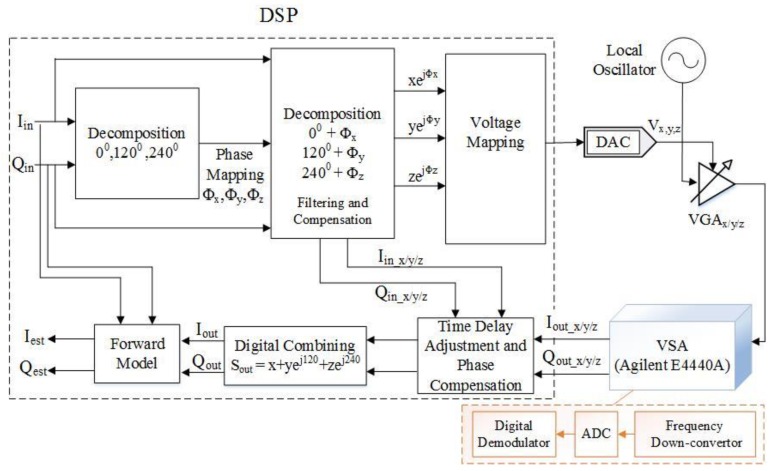
Block schematic of the mixer-less three-way amplitude modulator-based transmitter, branch-by-branch digital combining with signal processing.

Figure 5 shows the block schematic of the transmitter architecture using analog combining along with the digital signal processing blocks. Figure 6 shows the block schematic of the transmitter architecture using digital combining along with digital signal processing blocks. Here, only a single digital-to-analog convertor (DAC) is used and the measurement is taken for each branch separately simply using a single DAC. 

A training sequence of 10,000 samples is used to extract the coefficients using the least square technique [12]. Coefficients are then applied to the whole input sequence of 100,000 samples in order to estimate the output. The normalized mean square error (NMSE) between the estimated and measured output is calculated to evaluate the performance of the model. NMSE is calculated as:

(21) $$\mathrm{NMSE}=10\log_{10}(\frac{1}{N}\sum_{n=1}^{N}\frac{{\left|Y_{\mathrm{measured}}(n)-Y_{\mathrm{modeled}}(n)\right|}^{2}}{{\left|Y_{\mathrm{measured}}(n)\right|}^{2}})$$

where *Y*_measured_(*n*) is the actual measured output and *Y*_modeled_(*n*) is the estimated output obtained from the modified memory polynomial black box model.

## 4. Implementation of Mixer-Less Three-way Amplitude Modulator-Based Transmitter

**Figure 7:**
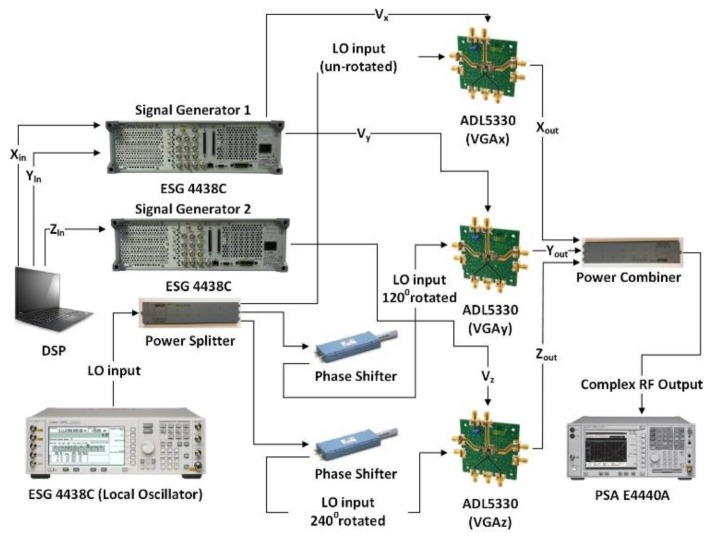
Implementation of the mixer-less three-way amplitude modulator-based transmitter using phase shifters and analog combining.

The mixer-less three-way amplitude modulator-based transmitter is implemented using three analog VGAs (ADL5330, Analog Devices Inc., Norwood, MA, USA) [22]. The specifications of the ADL5330 are given in Table 4. The evaluation of all three boards are depicted in Figure 7. All the VGAs are powered by a 5 V DC supply. 

Advanced Design System (ADS) is used to generate the complex baseband I/Q data. This complex baseband I/Q signal is decomposed into three envelopes using three coordinate decomposition algorithms and then mapped to control voltages in MATLAB. The three control voltages are then downloaded to two different signal generators (ESG4438C) as each signal generator has only two baseband outputs. Therefore, one signal generator is used for the generation of the control voltages Vx and Vy, while the second signal generator is used for the generation of control voltage Vz. Both signal generators are operated in synchronization. 

Another signal generator (ESG4438C) is used as the local oscillator (LO). The signal generators and the LO are triggered in synchronization. The analog gain control voltage Vx at the baseband output of the ESG-1 is sent to the gain control pin of VGAx. A similar procedure is used for VGAy and VGAz, respectively. The LO is provided to the three-way power divider (MACOM PN2090-6304-00) which has a loss of 7 dB in all three branches and has a frequency range from 0.5 GHz to 18 GHz. The LO at the first output port of the power divider is fed to the RF input port of VGAx. The LO at the second output port of the power divider is fed to the phase shifter (ARRA 9428A) with a frequency range from DC to 18 GHz, which rotates the LO by 120°. The output is then fed to the RF input port of VGAy. The LO at the third output port of the power divider is fed to the phase shifter (ARRA 9428A) which rotates the LO by 240°. The output of the second phase shifter is fed to the RF input port of VGAz. The RF outputs Vx, Vy, and Vz are summed together using a power combiner (MACOM PN2090-6304-00) to obtain a complex RF signal which is captured and digitized using a spectrum analyzer (PSA E4440A) and VSA software, respectively. The time alignment is carried out using the maximum correlation technique [23]. In order to retrieve the I/Q data from the captured signal, further signal processing is carried out in MATLAB. 

**Figure 8:**
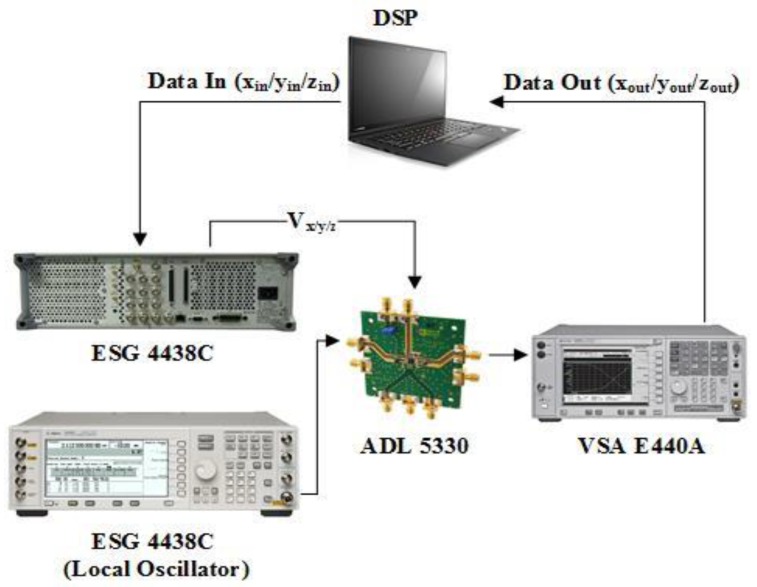
Branch-by-branch implementation of a mixer-less three-way amplitude modulator-based transmitter using digital combining.

Finally, Figure 8 shows the branch-by-branch implementation of mixer-less three-way amplitude modulator-based transmitter. In this setup the three VGAs are operated separately. The three voltages are generated in MATLAB and fed to the gain control pin of the VGAs one after the other. The LO is sent to the RF input port of the VGAs. The RF output of each VGA is captured separately using a power spectrum analyzer and digitized using VSA software. Time alignment is carried out distinctly on each branch using the maximum correlation technique. Finally, phase rotation and the power combining operations are performed in the digital domain.

## 5. Measurement Results

An LTE signal with a QPSK constellation is generated using ADS software to validate the proposed modeling technique and to evaluate the performance of the proposed methodology. The LTE signal is oversampled by a factor of 16 and the corresponding baseband signal has 100,000 samples which are sampled at a rate of 30.72 Msamples/sec. The complex baseband signal is then subjected to decomposition and processing. Components obtained after decomposition and digital processing are then mapped to control voltages and fed to the experimental setup as described in the previous section. The LO signal is sent at 2.2 GHz, having a power level of −3 dB. The signal is captured by a power spectrum analyzer and demodulated by VSA software followed by time alignment. The input to the proposed modified memory polynomial black box model is the original complex baseband I/Q signal and the output is the complex signal captured from the output of the transmitter. After obtaining the required input and output signals, model identification is performed to acquire the modeling coefficients and, finally, the modeled output. NMSE is then calculated between modeled output and measured output signals. The summary of results of the performance evaluation of the branch-by-branch digital combining mixer-less three-way amplitude modulator-based transmitter and analog combining mixer-less three-way amplitude modulator-based transmitter is depicted in Table 5 and Table 6, respectively. In terms of comparison, [20] shows a training NMSE of around −41 dB for the reverse model using a 1.4 MHz signal with digital combining. The proposed model allows obtaining a similar training NMSE for a forward model of the full three-way transmitter including the impairments of the analog combiner.

**Figure 9:**
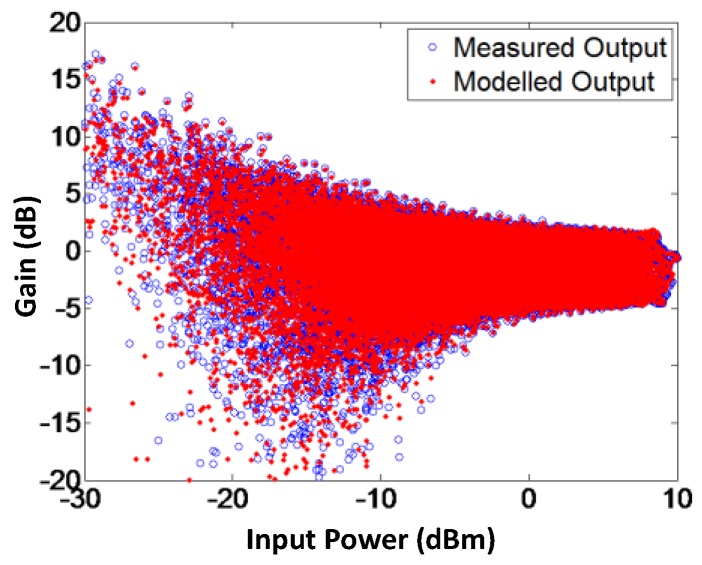
AM-AM characteristics of modeled and measured output signals for the branch-by-branch digital combining architecture. The modeled gain is capable of imitating the actual gain of the transmitter.

**Figure 10:**
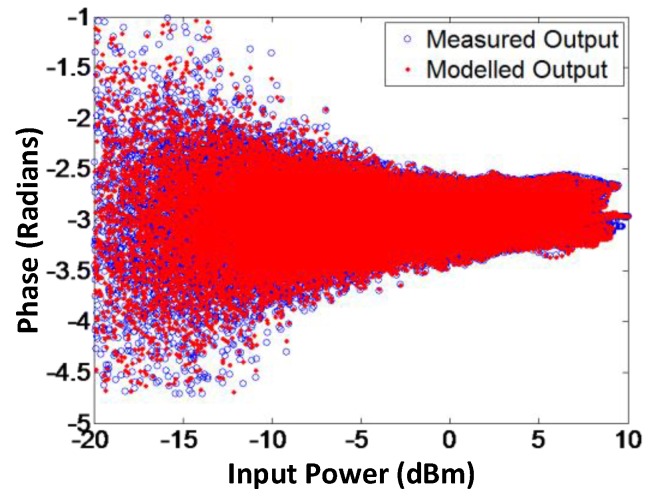
AM-PM characteristics of modeled and measured output signals for the branch-by-branch digital combining architecture, showing that the proposed technique is able to model the phase response efficiently.

**Figure 11:**
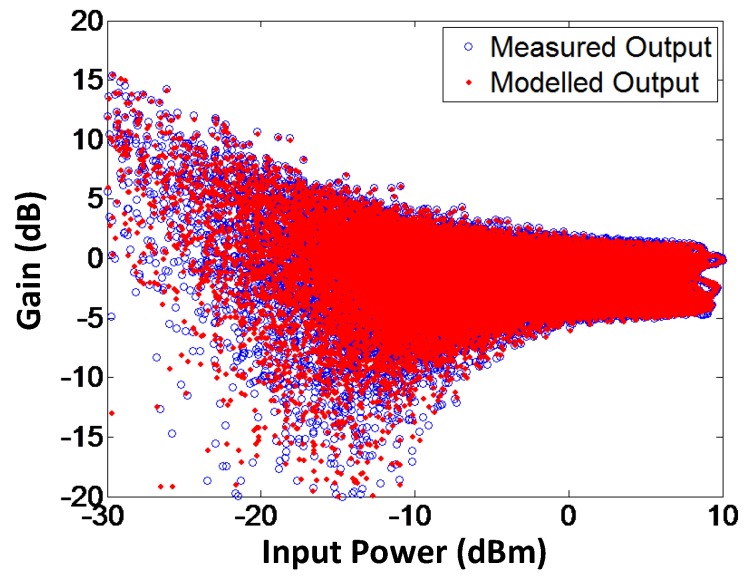
AM-AM characteristics of modeled and measured output signals for the analog combining architecture. Similar to when digital combining is used, the proposed method works effectively for modeling the gain response of the transmitter using analog combining.

**Figure 12:**
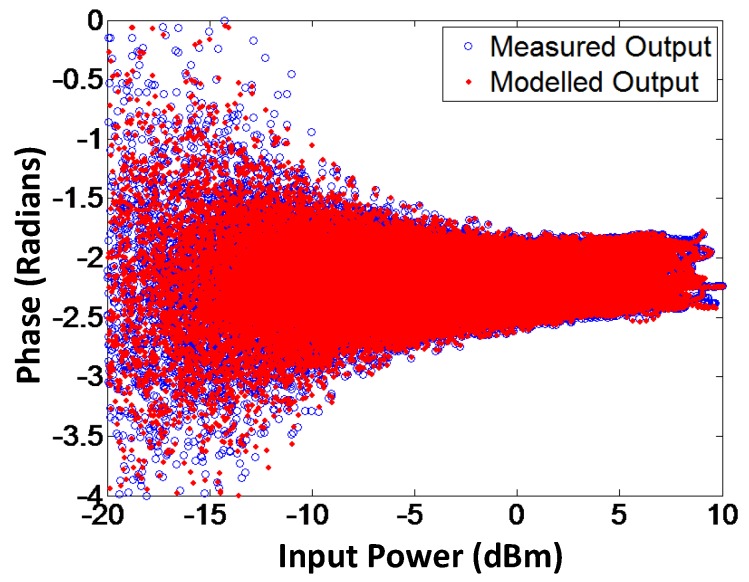
AM-PM characteristics of modeled and measured output signals for the analog combining architecture. The figure illustrates that the proposed augmented memory polynomial-based method is capable of modeling the phase response of the transmitter for analog combining.

**Figure 13:**
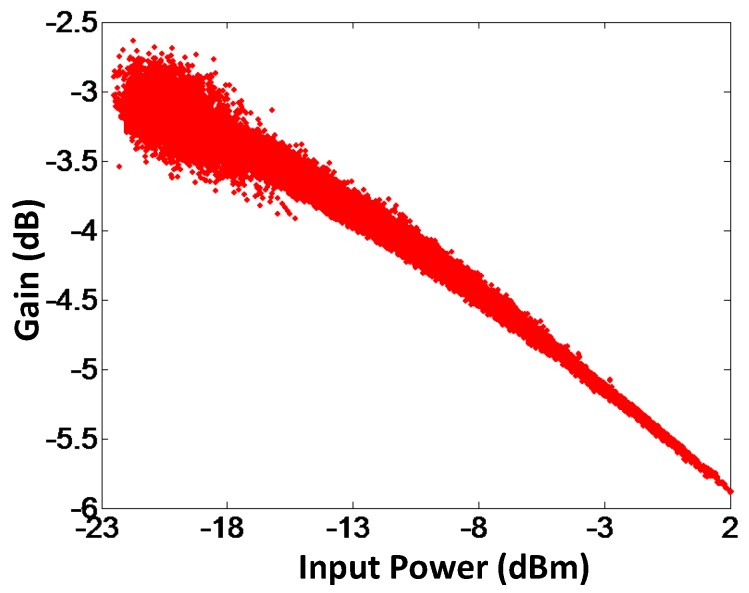
AM-AM characteristics of a single VGA.

**Figure 14:**
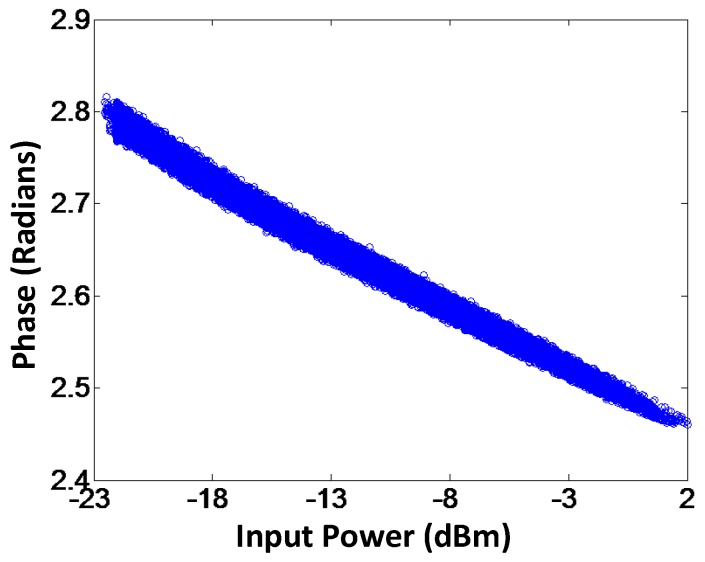
AM-PM characteristics of a single VGA.

To validate the proposed model, AM-AM and AM-PM of the modeled output and the measured output signals for branch-by-branch digital combining transmitter architecture are demonstrated in Figure 9 and Figure 10, respectively. In order to corroborate the three-way transmitter architecture and the model, the AM-AM and AM-PM of the modeled output and measured output signals for analog combining are shown in Figure 11 and Figure 12, respectively. Since the AM-AM and AM-PM plots are resultant of all three VGAs, the non-linearity of the single VGA cannot be observed. In order to observe the non-linearity exhibited by single VGA, the measured AM-AM and AM-PM responses of a single VGA are shown in Figure 13 and Figure 14, respectively.

**Figure 15:**
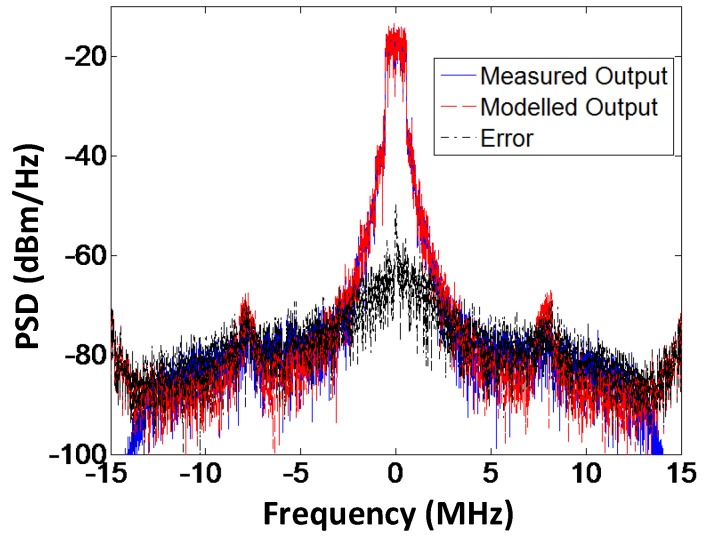
Spectral response of the modeled and measured output along with the error signal for the branch-by-branch digital combining architecture. The modeled output spectrum is closer to the measured spectrum leading to reduced error.

**Figure 16:**
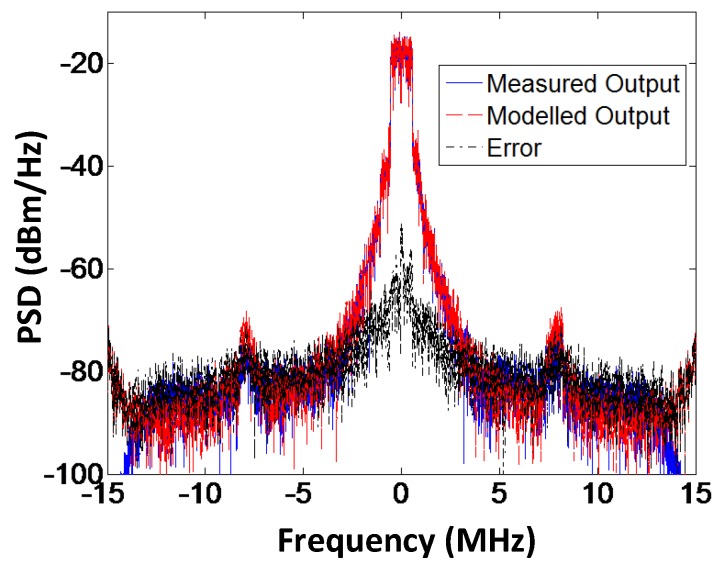
Spectral response of the modeled and measured output along with the error signal for the analog combining architecture. The proposed method is capable of reducing the modeling error even for analog combining.

The spectral response of the modeled output, the measured output, and the error signal for the branch-by-branch digital combining architecture and analog combining architecture are shown in Figure 15 and Figure 16, respectively. From the graphs we can deduce that the proposed forward model works exceptionally well for the three-way transmitter architecture. The measurement results for the performance evaluation of the three-way amplitude modulator-based transmitter for different bandwidths of the LTE signal are summarized in Table 7. The LTE signal with a 5 MHz bandwidth is oversampled by a factor of 48 and the baseband signal has 100,000 samples, sampled at a rate of 92.16 Msamples/s. The LO signal has a frequency of 2.2 GHz and a power level of −3 dBm. Bandwidth on the gain control of the VGA is limited to 3 MHz as seen from Table 4. However, the three-way architecture of transmitter and the proposed black box model works well with the 5 MHz bandwidth of the LTE signal, which proves that model and transmitter topology is not limited to the bandwidth of the LTE signal.

## 6. Conclusions

Distortion associated with conventional transmitters due to the use of imperfect mixers are nullified using the three-way transmitter topology as it employs VGAs for up-conversion. The three-way mixer-less topology of the transmitter does not use any RF band filtering at its output. In comparison with the conventional transmitter architecture, the absence of filters and wide RF bandwidth of the VGAs, power combiners, and phase shifters make this transmitter topology more reconfigurable and suitable for wideband applications.

A novel augmented memory polynomial forward model is proposed to model the characteristics of the three-way transmitter architecture and is validated using laboratory measurements. The performance of the proposed model, evaluated in terms of various figures of merits, shows the enhanced modeling capability. In addition, it is closer to the practical scenario as compared to its branch-by-branch counterpart [20].

## Figures and Tables

**Table 1:** Value of constants when 0° < *θ* < 120°.

Constants for $x_{\mathrm{in},1}$	Constants for $y_{\mathrm{in},1}$	Constants for $z_{\mathrm{in},1}$
$A_{1}=\frac{\sin^{3}(\theta -120{}^{\circ})}{\sin^{3}(\theta -120{}^{\circ})-\sin^{3}(\theta )}$	$A_{2}=\frac{-\sin^{3}(\theta )}{\sin^{3}(\theta -120{}^{\circ})-\sin^{3}(\theta )}$	$A_{3}=0$
$B_{1}=\frac{\sin^{2}(\theta -120{}^{\circ})\sin(\theta )}{\sin^{3}(\theta -120{}^{\circ})-\sin^{3}(\theta )}$	$B_{2}=\frac{-\sin^{2}(\theta -120{}^{\circ})\sin(\theta )}{\sin^{3}(\theta -120{}^{\circ})-\sin^{3}(\theta )}$	$B_{3}=0$
$C_{1}=\frac{\sin(\theta -120{}^{\circ})\sin^{2}(\theta )}{\sin^{3}(\theta -120{}^{\circ})-\sin^{3}(\theta )}$	$C_{2}=\frac{-\sin(\theta -120{}^{\circ})\sin^{2}(\theta )}{\sin^{3}(\theta -120{}^{\circ})-\sin^{3}(\theta )}$	$C_{3}=0$

**Table 2:** Value of constants when 120° < *θ* < 240°.

Constants for $x_{\mathrm{in},1}$	Constants for $y_{\mathrm{in},1}$	Constants for $z_{\mathrm{in},1}$
$A_{4}=0$	$A_{5}=\frac{\sin^{3}(\theta -240{}^{\circ})}{\sin^{3}(\theta -240{}^{\circ})-\sin^{3}(\theta -120{}^{\circ})}$	$A_{6}=\frac{-\sin^{3}(\theta -120{}^{\circ})}{\sin^{3}(\theta -240{}^{\circ})-\sin^{3}(\theta -120{}^{\circ})}$
$B_{4}=0$	$B_{5}=\frac{\sin^{2}(\theta -240{}^{\circ})\sin(\theta -120{}^{\circ})}{\sin^{3}(\theta -240{}^{\circ})-\sin^{3}(\theta -120{}^{\circ})}$	$B_{6}=\frac{-\sin^{2}(\theta -240{}^{\circ})\sin(\theta -120{}^{\circ})}{\sin^{3}(\theta -240{}^{\circ})-\sin^{3}(\theta -120{}^{\circ})}$
$C_{4}=0$	$C_{5}=\frac{\sin(\theta -240{}^{\circ})\sin^{2}(\theta -120{}^{\circ})}{\sin^{3}(\theta -240{}^{\circ})-\sin^{3}(\theta -120{}^{\circ})}$	$C_{6}=\frac{-\sin(\theta -240{}^{\circ})\sin^{2}(\theta -120{}^{\circ})}{\sin^{3}(\theta -240{}^{\circ})-\sin^{3}(\theta -120{}^{\circ})}$

**Table 3:** Value of constants when 240° < *θ* < 360°.

Constants for $x_{\mathrm{in},1}$	Constants for $y_{\mathrm{in},1}$	Constants for $z_{\mathrm{in},1}$
$A_{7}=\frac{-\sin^{3}(\theta -240{}^{\circ})}{\sin^{3}(\theta -360{}^{\circ})-\sin^{3}(\theta -240{}^{\circ})}$	$A_{8}=0$	$A_{9}=\frac{\sin^{3}(\theta -360{}^{\circ})}{\sin^{3}(\theta -360{}^{\circ})-\sin^{3}(\theta -240{}^{\circ})}$
$B_{7}=\frac{-\sin^{2}(\theta -360{}^{\circ})\sin(\theta -240{}^{\circ})}{\sin^{3}(\theta -360{}^{\circ})-\sin^{3}(\theta -240{}^{\circ})}$	$B_{8}=0$	$B_{9}=\frac{\sin^{2}(\theta -360{}^{\circ})\sin(\theta -240{}^{\circ})}{\sin^{3}(\theta -360{}^{\circ})-\sin^{3}(\theta -240{}^{\circ})}$
$C_{7}=\frac{-\sin(\theta -360{}^{\circ})\sin^{2}(\theta -240{}^{\circ})}{\sin^{3}(\theta -360{}^{\circ})-\sin^{3}(\theta -240{}^{\circ})}$	$C_{8}=0$	$C_{9}=\frac{\sin(\theta -360{}^{\circ})\sin^{2}(\theta -240{}^{\circ})}{\sin^{3}(\theta -360{}^{\circ})-\sin^{3}(\theta -240{}^{\circ})}$

**Table 4:** Specification of the VGA (ADL5330).

Specification	Value
Bandwidth on the gain control pin	3 MHz
Gain Range	60 dB
Control voltage range	0–1.4 V
Operating frequency	10 MHz–3 GHz
Linear-in-dB gain control function	20 mV/dB

**Table 5:** Summary of the performance evaluation of the branch-by-branch, digital combining, mixer-less three-way amplitude modulator-based transmitter.

Specification	Value
Signal bandwidth (MHz)	1.4
Number of testing samples	100,000
Number of training samples	10,000
Training NMSE (dB)	−39.56
Testing NMSE (dB)	−36.41
Non-linearity order and Memory depth	*K* = 3, *M* = 2

**Table 6:** Summary of the performance evaluation of the analog combining, mixer-less three-way amplitude modulator-based transmitter.

Specification	Value
Signal bandwidth (MHz)	1.4
Number of testing samples	100,000
Number of training samples	10,000
Training NMSE (dB)	−40.97
Testing NMSE (dB)	−36.90
Non-linearity order and Memory depth	*K* = 3, *M* = 2

**Table 7:** Summary of performance evaluation of different architectures for LTE signals with a 5 MHz bandwidth.

Specification	Digital Combining Architecture	Analog Combining Architecture
Signal bandwidth (MHz)	5	5
Number of testing samples	100,000	100,000
Number of training samples	10,000	10,000
Training NMSE (dB)	−34.48	−34.56
Testing NMSE (dB)	−31.93	−32.08
Non-linearity order and Memory depth	*K* = 3, *M* = 2	*K* = 3, *M* = 2

## Appendix A

**Calculation Details for the Forward Model Expression When *S*_in_ Lies in 0°–120°**

Since the output of a single VGA can be modeled with a memory polynomial, the outputs *x_out_*, *y_out_*, and *z_out_* of the three VGAs VGAx, VGAy, or VGAz, respectively, can be modeled as follows:

(A1) $$x_{\mathrm{out}}(n)=\sum_{k=1}^{K_{x}}\sum_{m=0}^{M_{x}}h_{k,m}^{x}{\left(x_{\mathrm{in},1}(n-m)\right)}^{k}$$

(A2) $$y_{\mathrm{out}}(n)=\sum_{k=1}^{K_{y}}\sum_{m=0}^{M_{y}}h_{k,m}^{y}{\left(y_{\mathrm{in},1}(n-m)\right)}^{k}$$

(A3) $$z_{\mathrm{out}}(n)=\sum_{k=1}^{K_{z}}\sum_{m=0}^{M_{z}}h_{k,m}^{z}{\left(z_{\mathrm{in},1}(n-m)\right)}^{k}$$

where, $h_{k,m}^{x}$, $h_{k,m}^{y}$, and $h_{k,m}^{z}$ are the complex model coefficients of the memory polynomials modelling VGAx, VGAy, and VGAz, respectively. (*K_x_*, *M_x_*), (*K_y_*, *M_y_*), and (*K_z_*, *M_z_*) are nonlinearity orders and memory depths of the memory polynomials modelling VGAx, VGAy, and VGAz, respectively. After, replacing the values of *x*_in,1_, *y*_in,1_, and *z*_in,1_ by their expressions from Equations (4)–(6) and after applying binomial development, (A1)–(A3) can be written as:

(A4) $$x_{\mathrm{out}}=\sum_{k=1}^{K}\sum_{m=0}^{M}\sum_{i_{1}+i_{2}+i_{3}=k}h_{k,m}^{x}\alpha_{i_{1}i_{2}i_{3}}A_{1}^{i_{1}}B_{1}^{i_{2}}C_{1}^{i_{3}}S_{1}^{i_{1}}S_{2}^{i_{2}}S_{3}^{i_{3}}$$

(A5) $$y_{\mathrm{out}}=\sum_{k=1}^{K}\sum_{m=0}^{M}\sum_{i_{1}+i_{2}+i_{3}=k}h_{k,m}^{y}\beta_{i_{1}i_{2}i_{3}}A_{2}^{i_{1}}B_{2}^{i_{2}}C_{2}^{i_{3}}S_{1}^{i_{1}}S_{2}^{i_{2}}S_{3}^{i_{3}}$$

(A6) $$z_{\mathrm{out}}=\sum_{k=1}^{K}\sum_{m=0}^{M}\sum_{i_{1}+i_{2}+i_{3}=k}h_{k,m}^{z}\gamma_{i_{1}i_{2}i_{3}}A_{3}^{i_{1}}B_{3}^{i_{2}}C_{3}^{i_{3}}S_{1}^{i_{1}}S_{2}^{i_{2}}S_{3}^{i_{3}}$$

where $\alpha_{i_{1}i_{2}i_{3}}$, $\beta_{i_{1}i_{2}i_{3}}$, and $\gamma_{i_{1}i_{2}i_{3}}$ are constants resulting from the additions of binomial constants. Since the output is obtained by summing the three VGAs’ outputs as follows:

(A7) $$S_{\mathrm{out}}=x_{\mathrm{out}}+y_{\mathrm{out}}e^{j120^{\circ }}+z_{\mathrm{out}}e^{j240^{\circ }}$$

By replacing *x*_out_, *y*_out_, and *z*_out_ by their expressions from (A4)–(A6), we obtain:

(A8) $$S_{\mathrm{out}}=\sum_{k=1}^{K}\sum_{m=0}^{M}\sum_{i_{1}+i_{2}+i_{3}=k}\left[\begin{array}{l} H_{k,m,i_{1}i_{2}i_{3}}^{x}A_{1}^{i_{1}}B_{1}^{i_{2}}C_{1}^{i_{3}}+\\ H_{k,m,i_{1}i_{2}i_{3}}^{y}A_{2}^{i_{1}}B_{2}^{i_{2}}C_{2}^{i_{3}}e^{j120{}^{\circ}}+\\ H_{k,m,i_{1}i_{2}i_{3}}^{z}A_{3}^{i_{1}}B_{3}^{i_{2}}C_{3}^{i_{3}}e^{j240{}^{\circ}} \end{array}\right]S_{1}^{i_{1}}S_{2}^{i_{2}}S_{3}^{i_{3}}$$

where $H_{k,m,i_{1}i_{2}i_{3}}^{x}=h_{k,m}^{x}\alpha_{i_{1}i_{2}i_{3}}$, $H_{k,m,i_{1}i_{2}i_{3}}^{y}=h_{k,m}^{y}\beta_{i_{1}i_{2}i_{3}}$, and $H_{k,m,i_{1}i_{2}i_{3}}^{z}=h_{k,m}^{z}\gamma_{i_{1}i_{2}i_{3}}$. As seen from Table 1, we know that *B*_1_ = −*B*_2_ and *C*_1_ =−*C*_2_, while values of *A*_1_ and *A*_2_ are distinct. We also know that values of *A*_3_, *B*_3_ and *C*_3_ are 0 for this sector. Therefore, Equation (A8) can be represented as:

(A9) $$S_{\mathrm{out}}=\sum_{k=1}^{K}\sum_{m=0}^{M}\sum_{i_{1}+i_{2}+i_{3}=k}B_{1}^{i_{2}}C_{1}^{i_{3}}\left[H_{k,m,i_{1}i_{2}i_{3}}^{x}A_{1}^{i_{1}}+{\left(-1\right)}^{i_{2}+i_{3}}H_{k,m,i_{1}i_{2}i_{3}}^{y}A_{2}^{i_{1}}e^{i120{}^{\circ}}\right]S_{1}^{i_{1}}S_{2}^{i_{2}}S_{3}^{i_{3}}$$

Now, by substituting the values of *A*_1_, *A*_2_, *B*_1_, and *C*_1_ from Table 1, we obtain:

(A10) $$S_{\mathrm{out}}=\sum_{k=1}^{K}\sum_{m=0}^{M}\sum_{i_{1}+i_{2}+i_{3}=k}D_{1}^{-k}\left[\begin{array}{c} H_{k,m,i_{1}i_{2}i_{3}}^{x}sin^{i_{2}+2i_{3}}(\theta )sin^{3i_{1}+2i_{2}+i_{3}}(\theta -120{}^{\circ})+\\ {\left(-1\right)}^{k}H_{k,m,i_{1}i_{2}i_{3}}^{y}sin^{3i_{1}+i_{2}+2i_{3}}(\theta )sin^{2i_{2}+i_{3}}(\theta_{1}-120{}^{\circ})e^{j120{}^{\circ}} \end{array}\right]S_{1}^{i_{1}}S_{2}^{i_{2}}S_{3}^{i_{3}}$$

where *D*_1_ represents the denominator in 0°–120°, and can be represented as:

(A11) $$D_{1}=\sin^{3}(\theta -120{}^{\circ})-\sin^{3}(\theta )$$

leading to the final expression shown in Equation (18) shown below:

(A12) $$S_{\mathrm{out}}(n)=\sum_{k=1}^{K}\sum_{m=0}^{M}\sum_{p_{1}+p_{2}=3k}G_{k,m,p_{1},p_{2}}\frac{\sin^{p_{1}}(\theta )\sin^{p_{2}}(\theta -120{}^{\circ})}{{\left(\sin^{3}(\theta -120{}^{\circ})-\sin^{3}(\theta )\right)}^{k}}S_{\mathrm{in}1}{}^{k}(n-m)$$

where $G_{k.m,p_{1},p_{2}}\;$are constants obtained by adding the terms $H_{k,m,i_{1}i_{2}i_{3}}^{y}$ and ${\left(-1\right)}^{k}H_{k,m,i_{1}i_{2}i_{3}}^{z}$ corresponding to the same exponents $p_{1}$ and $p_{2}$ of the term $\sin^{p_{1}}\left(\theta \right)\sin^{p_{2}}\left(\theta -120{}^{\circ}\right).$

Similar analysis can be done for the other two sectors. The outputs can then be obtained for 120° < *θ* < 240°:

(A13) $$S_{\mathrm{out}}(n)=\sum_{k=1}^{K}\sum_{m=0}^{M}\sum_{p_{1}+p_{2}=3k}G_{k,m,p_{1},p_{2}}^{\prime }\frac{\sin^{p_{1}}(\theta -120{}^{\circ})\sin^{p_{2}}(\theta -240{}^{\circ})}{{\left(\sin^{3}(\theta -240{}^{\circ})-\sin^{3}(\theta -120{}^{\circ})\right)}^{k}}S_{\mathrm{in}1}{}^{k}(n-m)$$

where $G_{k,m,p_{1},p_{2}}^{\prime }$ are constants from *A*_4_, *B*_4_, *C*_4_, *A*_5_, *B*_5_, *C*_5_, *A*_6_, *B*_6_, and *C*_6_ similarly to $\;G_{k.m,p_{1},p_{2}}.$

For 240° < *θ* < 360°:

(A14) $$S_{\mathrm{out}}(n)=\sum_{k=1}^{K}\sum_{m=0}^{M}\sum_{p_{1}+p_{2}=3k}G_{k,m,p_{1},p_{2}}^{\prime \prime }\frac{\sin^{p_{1}}(\theta -240{}^{\circ})\sin^{p_{2}}(\theta -360{}^{\circ})}{{\left(\sin^{3}(\theta -360{}^{\circ})-\sin^{3}(\theta -240{}^{\circ})\right)}^{k}}S_{\mathrm{in}1}{}^{k}(n-m)$$

where $\;G_{k,m,p_{1},p_{2}}^{\prime \prime }$ are constants from *A*_7_, *B*_7_, *C*_7_, *A*_8_, *B*_8_, *C*_8_, *A*_9_, *B*_9_, and *C*_9_, similarly to $G_{k,m,p_{1},p_{2}}.$
